# A prospective randomized comparison of early embryo cleavage kinetics between two media culture systems

**DOI:** 10.12669/pjms.326.10809

**Published:** 2016

**Authors:** Huan Zhang, Yi Zheng, Yonggen Wu, Danna Ye, Xuefeng Huang

**Affiliations:** 1 Huan Zhang, MD, Department of Reproductive Medical Center, The First Affiliated Hospital of Wenzhou Medical University, Wenzhou, Zhejiang, People’s Republic of China; 2 Yi Zheng, MD, Department of Reproductive Medical Center, The First Affiliated Hospital of Wenzhou Medical University, Wenzhou, Zhejiang, People’s Republic of China; 3 Yonggen Wu, MD, Department of Reproductive Medical Center, The First Affiliated Hospital of Wenzhou Medical University, Wenzhou, Zhejiang, People’s Republic of China; 4 Danna Ye, MD, Department of Reproductive Medical Center, The First Affiliated Hospital of Wenzhou Medical University, Wenzhou, Zhejiang, People’s Republic of China; 5 Xuefeng Huang, PhD, Department of Reproductive Medical Center, The First Affiliated Hospital of Wenzhou Medical University, Wenzhou, Zhejiang, People’s Republic of China

**Keywords:** Time-lapse imaging, Culture media, Embryonic development, Morphokinetics

## Abstract

**Objective:**

To investigate whether early embryo cleavage kinetics were affected by type of culture media.

**Methods:**

In this prospective sibling-split study, 620 oocytes from 37 patients were randomly allocated into two groups: Cook group and Vitrolife group. Oocytes/embryos in Cook group, would be cultured with Cook sequential culture medium, while oocytes/embryos in Vitrolife group, would be cultured with Vitrolife sequential culture medium. Time-lapse imaging technology was used to calculate exact timing of early embryo cleavage events which included time to 2PN breakdown, cleavage to 2-, 3-, 4-, 5- cell and the time duration in the 2-,3-cell stage. Then these timing of early embryo cleavage events were compared between Cook group and Vitrolife group. Moreover, fertilization rate, cleavage rate, high quality embryo rate, usable blastocyst rate, pregnancy rate and implantation rate of these two groups were also analyzed.

**Results:**

The results showed there were no differences in all timing of early embryo cleavage events between the two groups. In addition, the two groups were similar in fertilization rate (Cook 71.0% vs. Vitrolife 71.3%, P>0.05), cleavage rate (Cook 98.1% vs. Vitrolife 98.2%, P>0.05), high quality embryo rate (Cook 52.1% vs. Vitrolife 52.7%, P>0.05), usable blastocyst rate (Cook 29.7% vs. Vitrolife 28.0%, P>0.05), pregnancy rate (Cook 46.7% VS. Vitrolife 50.0%, P>0.05) and implantation rate (Cook 30.3% VS. Vitrolife 29.0%, P>0.05).

**Conclusions:**

Morphokinetics used for embryo selection are not affected by the two different culture media.

## INTRODUCTION

With the development of assisted reproductive technology, some new tools were used to improve clinical outcomes. In comparison with morphological assessment, time-lapse imaging technology is a new method for *in vitro* fertilization (IVF) laboratories. It is a non-invasive method that capture the images of dynamic embryonic development and increase the information of cell division kinetics.^1^-^3^ Besides, time-lapse devices could offer the possibility for 24-hours monitoring, without disturbing the culture conditions.^4^,^5^ Models base on morphokinetics of early embryonic development in combination with morphological assessment have been shown to improve clinical outcomes.^6^-^9^ Therefore it is reasonable to assume that morphokinetics of early embryonic development will substantially provide more information about embryo viability. However, early cleavage kinetics may affected by some potential factors, such as the type of media which supply energy substances and other elements for embryonic development.^10^

Nowadays, commercial media have gradually replaced ‘in-house’ prepared media.^11^ In contrast to ‘in-house’ prepared media, commercially available media could avoid the potential disadvantages, including difficulties in quality control and labor-intense preparation.^12^ However different commercial media possess different composition and would result in the differences of culture conditions, which are crucial to embryonic development, implantation and a healthy pregnancy.^13^ So far, the research about commercial media were focusing on whether the type of commercial media have effect on day two or day three morphology, implantation, pregnancy rates and birth-weight of the fetus.^14^,^15^ Little was known about the relationship between early embryo cleavage kinetics and the type of commercial media.^16^

In the present study, we evaluated the effect of two types of commercial culture media on early cleavage kinetics of embryonic development in sibling oocytes and expected to investigate whether implantation prediction models based on time-lapse analysis could be used in various embryo culture systems.

## METHODS

This is a prospective sibling-split study, performed between January and December in 2014. Inclusion criteria for this study were as follows: (1) the patients were aged <45 years; (2) body mass index (BMI) of patients was <35.0 kg/m^2^; (3) Male partners had a sperm concentration ≥ 10×10^6^/ml and motility ≥30% in raw semen. In addition, exclusion criteria for this study were as follows: (1) women with known previous poor ovarian response to ovarian stimulation, endometriosis, polycystic ovary syndrome (PCOS), hydrosalpynx, uterine pathology; (2) cycles with less than 8 oocytes.

All patients underwent controlled ovarian stimulation. When at least one follicle measured 18 mm or more, Human chorionic gonadotropin (HCG, Livzon, China) was administered. Oocyte retrieval was performed 36 hours after HCG under ultrasound guidance.

After follicle aspiration, the oocytes were randomly distributed into one of the two groups according to a randomization table: in case of Cook group, the oocytes were put into a dish filled with K-SIFM medium (Cook, Australia), while Vitrolife group oocytes were put into a dish filled with G-IVF Plus medium (Vitrolife, Sweden). Four hours later, oocytes were cultured in 80 µl droplets of G-IVF Plus medium (Vitrolife group) or K-SIFM medium (Cook group) and inseminated with approximately 40, 000 progressively motile spermatozoa. After 16–18 hours, fertilization was verified then embryos were placed in Primo Vision embryo culture dish that were filled with K-SICM Medium (Cook group) or G1 Plus Medium (Vitrolife group). These dishes were then loaded into the Primo Vision system (Vitrolife, Sweden), which is a compact digital inverted microscope system designed to be placed inside of incubators under the condition of 37°C, 5% O_2_ and 6%CO_2_.

The exact time of the embryonic development events was calculated in hours after insemination as described by Meseguer et al.^17^ Images of each embryo were taken every five minutes in seven different focal planes during 72 hours of culture. The term pronuclei breakdown (PNF) defined as when both two PN disappeared. While t2, t3, t4, t5 defined as the time when 2-,3-,4-,5- cell were observed the first time, respectively. We also define cc2 as the time duration in the two-cell stage (cc2 =t3- t2), and s2 corresponded to the time duration in the three-cell stage (s2 = t4 - t3).

On the morning of day 3, embryo transfer was performed under abdominal ultrasound guidance. The two or three transferred embryos were according to their morphology first, and then selected according to morphokinetics analysis. Surplus embryos were frozen or transferred from cleavage medium (K-SICM, Cook, Australia or G1 Plus, Vitrolife, Sweden) to blastocyst medium (K-SIBM, Cook, Australia or G2 Plus, Vitrolife, Sweden).

Four weeks later, clinical pregnancy was identified by development of a gestational with fetal heart beat on ultrasound examination.

## RESULTS

In this study, 620 oocytes were retrieved from 37 patients. The clinical characteristics of patients, are listed in Table-I. According to our procedure, 303 oocytes were allocated into Cook group, and 317 oocytes were allocated into Vitrolife group. There were no differences in fertilization rate (71.0% vs. 71.3%, P>0.05) and cleavage rate (98.1% vs. 98.2%, P>0.05) between Cook group and Vitrolife group.

**Table-I:** Characteristics of patients

Variables	
Number of patients	37
Female age (y)	31.98 ± 4.14
Basal LH (IU/L)	5.31 ± 2.19
Basal E2 (pmol/L)	157.47 ± 91.68
Basal FSH (IU/L)	7.15 ± 1.83
Basal BMI (kg/m2)	20.37 ± 2.17
Endometrial thickness on day of HCG (mm)	10.18 ± 2.13
Duration of infertility (y)	3.03 ± 3.01
No. of oocytes retrieved per patient	16.97 ± 5.56

In this study, immature oocytes and the oocytes which were not fertilized normally or did not complete the first division were excluded in time-lapse morphokinetics analysis. All the timing of time-lapse parameters (from PNF to t5) of the two groups are shown in Table-II. Statistically non-significant differences in timing of early embryo cleavage kinetics were found between the two groups. We also compared the percentage of embryos falling within the optimal ranges proposed for t2, t3, t5, cc2 and s2, the results showed that there were no significant differences between the two groups (Table-III).

**Table-II:** Timings of early embryo cleavage kinetics were compared according to the media utilized.

Variable	Culture media	N	Mean	CI 95%		P value

				Lower limit	Upper limit	
PNF	Cook	211	25.04	24.54	25.53	0.836
	Vitrolife	222	25.10	24.68	25.53	
t2(h)	Cook	211	28.56	27.82	29.30	0.610
	Vitrolife	222	28.30	27.65	28.95	
t3(h)	Cook	203	37.12	36.21	38.02	0.837
	Vitrolife	219	37.00	36.22	37.77	
t4(h)	Cook	196	39.48	38.60	40.36	0.467
	Vitrolife	210	39.05	38.29	39.82	
t5(h)	Cook	184	50.54	49.35	51.73	0.332
	Vitrolife	193	49.75	48.66	50.83	
cc2(h)	Cook	203	8.66	7.90	9.42	0.834
	Vitrolife	219	8.76	8.12	9.41	
s2(h)	Cook	196	2.71	2.14	3.28	0.324
	Vitrolife	210	2.33	1.81	2.84	

**Table-III:** Percentage of embryos falling within optimal ranges proposed for t2, t3, t5, cc2 and s2.

	Cook	Vitrolife	P value
T2 (24.3-27.9 h)	55.0%	56.8%	0.709
T3 (35.4-40.3 h)	58.6%	58.9%	0.953
T5 (48.8–56.6 h)	60.3%	56.0%	0.390
cc2(≤11.9 h)	76.4%	81.7%	0.182
s2 (≤0.75 h)	37.8%	46.9%	0.063

On day 3, day 5 and day 6, morphology score was performed. There were no differences in high quality embryo rate (Cook 52.1% vs. Vitrolife 52.7%, P>0.05) and usable blastocyst rate (Cook 29.7% vs. Vitrolife 28.0%, P>0.05) between the two groups.

A total of 64 embryo was transferred. 15 cycles where the transferred embryos (n= 33) were only cultured in Cook media, while 14 cycles where the transferred embryos (n= 31) were only cultured in Vitrolife media. There were no differences in pregnancy rate (Cook 46.7% VS. Vitrolife 50.0%, P>0.05) and implantation rates (Cook 30.3% VS. Vitrolife 29.0%, P>0.05) between the two groups.

## DISCUSSION

This study suggested that embryos cultured in Cook culture media and Vitrolife culture media were similar in early embryo cleavage kinetics and clinical outcomes. It was confirmed by the data that these two culture systems have no differences in timing of morphokinetic events (form PNF to t5), day 3 high quality embryo rate, usable blastocyst rate, pregnancy rate and implantation rate.

It is difficult to assess the impact of culture media on embryonic development, because several potential confounding factors may affect the outcomes. Here, we optimized the experimental designs to minimize such factors. Firstly, we designed a sibling-split study, where oocytes were divided into two group, half oocytes were allocated into Cook media culture system and the others were allocated into Vitrolife media culture system. As well known, oocytes are generally derived from women who underwent controlled ovarian stimulation. The quality of oocytes are great variation in different women. By using sibling oocytes, we were able to eliminate individual differences. Secondly, culture conditions were identical for sibling oocytes/embryos during *in vitro* culture, thus to decrease the effect of variations in gas concentration, humidity and temperature. Therefore, we can evaluate the two media culture system as real as possible, since the confounding factors inherent in clinical embryology have been reduced.

Nowadays, several commercial culture media are available on market. Different culture media may lead to great difference in early embryonic development. This study chose to compare Cook media and Vitrolife media, due to that they were commonly used in a lot in laboratories, including our laboratory. Although the exact formulations of the two media have not been public, a research had determined the compositions of some commercially available culture media recently.^13^ One main difference between Cook media and Vitrolife media was found in the amino acid composition. Cook cleavage medium contains both essential and non-essential amino acids, while Vitrolife cleavage medium contains only non-essential amino acids. Amino acids are very essential element for embryonic development and embryo metabolic,^18^ it can be osmolytes, chelators, energy substrates, antioxidants, biosynthetic precursors and energy metabolism regulators.^19^ Besides, another main difference between the two culture media systems was found in the concentration of lactate and ratio of lactate/pyruvate. It was reported that pyruvate and lactate are the early embryo’s primary source of energy, the ratio of lactate/pyruvate would effect reductive-oxidative balance in the culture medium.^20^,^21^ So, whether the different composition of the two media would affect early embryo development need to be investigated.

Recently, some studies did compare these two media, a previous study showed that Vitrolife culture media group got higher pregnancy rate and implantation rate than Cook culture media group through analyzing 826 first IVF treatment cycles.^22^ In addition, Van Langendonckt also found Vitrolife sequential media resulted in a significant higher pregnancy rate as compared with the use of Cook media, in a subset of patients with at least five embryos put into culture.^23^ In contrast, another report presented opposite results that no difference were found between the two media, either in pregnancy rate, or in implantion rate.^24^ However, researches mentioned above had no conclusion which is better for embryo culture *in vitro* and their studies only focused on clinical outcomes, little was known about early embryo cleavage kinetics.

As well known, time-lapse enabled a more precise definition of events occurring during growth of embryos than static morphological assessment. Thus, it is an ideal tool to study the dynamic biological processes of embryo development. In the present study, we applied time-lapse technology to analysis some specific morphokinetic parameters, including PNF, t2, t3, t4, t5, cc2 and s2. The results showed there were no significant statistical differences in timing of these morphokinetic parameters between the two media groups.

Furthermore, it is worth mentioning that some morphokinetics parameters have been used to predict implantation.^17^,^25^ According to the study of Meseguer, the optimal range of t2, t3, t5, cc2, s2 were 24.3-27.9 h, 35.4-40.3 h, 48.8-56.6 h, ≤11.9h, ≤0.76 h, respectively.^17^ Embryos fall in the optimal range indicating higher viability. In this study, the percentage of embryo fell in the optimal range of t2, t3, t5, cc2, s2 were also compared respectively, and no significant statistical difference was found between the two media groups. So it can be understood that as the differences of the compositions between the two media had no effect on early embryo cleavage kinetics.

## CONCLUSION

This study suggested that embryos cultured in Cook culture media and Vitrolife culture media have similar early embryo cleavage kinetics.
